# Initial Radiographic Outcomes of Titanium Cage Implants in Anterior Cervical Discectomy and Fusion: A Retrospective Review

**DOI:** 10.7759/cureus.85350

**Published:** 2025-06-04

**Authors:** Mary J Parkash, Hushil S Sandhu, John Hamilton, Samantha Taylor, Rakesh Dhokia

**Affiliations:** 1 School of Medicine, Dentistry and Biomedical Sciences, Queen's University Belfast, Belfast, GBR; 2 Department of Trauma and Orthopaedics, Belfast Health and Social Care Trust, Belfast, GBR; 3 Dentistry and Biomedical Sciences, Queen's University Belfast, Belfast, GBR

**Keywords:** anterior cervical discectomy and fusion, cobb angle, intervertebral disc height, radiological outcome, titanium

## Abstract

Introduction

Anterior cervical discectomy and fusion (ACDF) is a common surgical treatment for degenerative diseases and trauma in the cervical spine. The procedure aims to restore and maintain lordosis in the cervical spine and increase intervertebral disc height; ultimately leading to vertebral body fusion and spinal stabilisation. Polyetheretherketone cages are commonly utilised; however, new implant technologies such as 3D-printed titanium cages have emerged. There are few published reports regarding titanium cages, and therefore, this study evaluated the initial radiological outcomes of the CONDUIT^TM ^EIT Cellular Titanium Cervical Cage (DePuy Synthes, Wurmlingen, Germany). The aim is to contribute to the existing literature and establish the non-inferiority of this implant type compared to other common implants.

Methods

This study reviewed 29 patients retrospectively, who underwent 36 ACDF procedures in the Regional Spinal Unit in Northern Ireland. Radiological images were assessed at three time points: pre-operative, one-day post-operative, and follow-up (on average 6.19 months later). At each time point, intervertebral disc height, segmental Cobb angle of the operated segment, and the overall C2-C7 Cobb angle of the cervical spine were measured. To ensure the accuracy of the assessed data, all images were independently reviewed by both the author and an external observer, allowing for the calculation of the intraclass correlation coefficient for each measurement. The Wilcoxon signed rank test was used to assess the significance of changes in each radiographic variable between different time points. Spearman’s rank correlation was used to identify any relationships between the radiographic variables.

Results

Intervertebral disc height increased from an average pre-operative value of 3.79 ±1.38 mm to an average follow-up value of 5.94 ± 1.41 mm, significantly increasing by an average of 2.14 ± 1.98mm (p<0.001). Segmental Cobb Angle increased from an average pre-operative kyphotic value of -1.14 ± 8.42° to an average follow-up lordotic reading of 1.71 ± 6.28°, significantly increasing by an average of 2.86 ± 7.60° (p=0.045). Overall, the C2-C7 Cobb angle increased from an average pre-operative lordotic value of 7.36 ± 8.46° to an average follow-up lordotic value of 10.34± 12.09°, significantly increasing by an average of 2.98 ± 9.94° (p=0.021). Interestingly, all variables were the highest immediately post-operatively and then declined over the follow-up period (intervertebral disc height declined by 0.62 ± 0.98mm (<0.001), segmental Cobb angle by 0.96 ± 1.39° (p<0.007) and overall Cobb angle by 0.29 ± 8.46° (p=0.422). There was a moderate positive Spearman’s correlation coefficient of 0.498 (p=0.002) between the change in intervertebral disc height and overall C2-C7 Cobb angle. Additionally, the correlation coefficient between change in intervertebral disc height and segmental Cobb angle was 0.238 (p=0.162), indicating a non-significant weak positive relationship. The correlation coefficient between overall Cobb angle and segmental Cobb angle was -0.17 (p=0.92), indicating a very weak negative relationship, although not significant.

Conclusions

Titanium cages demonstrated effectiveness in this study, showing favourable initial radiological outcomes and proving non-inferior to other cage types in this regard. Further research is needed to evaluate titanium cages, including their associated clinical outcomes, comprehensively.

## Introduction

Anterior cervical discectomy and fusion (ACDF) provides effective surgical management for patients presenting with cervical spine degenerative disease with myeloradiculopathy or cervical spine trauma. Anterior cervical discectomy and fusion was initially described by Smith et al., and their approach remains the gold standard today in many spinal units, including the Belfast Health and Social Care Trust (BHSCT), where this study was conducted [1].

Cervical spine anatomy

The cervical spine has a lordotic curvature of around 20-35° [2], with Yukawa et al. [3] determining C2-C7 lordosis as 13.9 ± 12.3°. One of the main ways of assessing cervical lordosis is the C2-C7 (overall) Cobb angle. This method involves drawing a line across the inferior endplate of C2 and another line across the inferior endplate of C7 on a lateral cervical X-ray: Perpendicular lines are drawn from each of these lines, and their angle of intersection is the angle of cervical curvature [4]. However, Harrison et al. suggest that often the Cobb angle can underestimate the actual degree of lordosis in individuals if the vertebral endplates are irregular, wedged, or diseased [4]. Yukawa et al. found that the Cobb angle was underestimated due to the C2 inferior endplate orientation, indicating the angle was larger than 13.9 ± 12.3° [3]. The spine maintains its lordotic curvature because of the wedge shape of the cervical vertebrae [5] and because the intervertebral discs are slightly thicker anteriorly [6]. Normal intravertebral cervical disc height ranges from 3.5-4.9 mm and decreases with age, with the highest degree of degeneration being at the C5-6 and C6-7 disc spaces [6]. Chronic disc herniation can lead to nerve compression in the intervertebral foramen, and furthermore, there is a reduction in cervical spine mechanical stability and loss of disc height [7].

Ferrara LA found that the loss of disc height leads to buckling of the spinal ligaments and annular fibres under compressive loads, which worsen with movement, and eventually the anterior aspect of the spine compresses [8]. Mechanical instability and uneven axial load cause a loss in cervical lordosis and put stress on the surrounding soft tissue and bone, eventually leading to compression of the spinal cord or nerve root [8].

CONDUIT^TM^ implant and its surgical application

The main goals of ACDF are to restore intervertebral height, improve cervical lordosis, and achieve intervertebral bony fusion to prevent spinal instability [9]. Additionally, decompression of the nerve root and or spinal cord is the key to relieving symptoms. Titanium cages and polyetheretherketone (PEEK) cages are most frequently used, and the ideal cage must meet the goals of surgery in achieving fusion and having the lowest subsidence rates possible [10]. Traditional titanium cages have a higher elasticity module than PEEK cages and hence might cause higher subsidence rates but they are proven to have good immediate cervical stability [11], and high rates of osteointegration and bone cell infiltration into the cages due to the fact they are hydrophilic and porous, therefore promoting fusion [10]. The CONDUIT^TM^ cervical implant (DePuy Synthes, Wurmlingen, Germany) resembles the shape of a dome to fit vertebral endplate anatomy and has a rough elevated surface engineered to allow for bone grafting. The plate itself possesses a lattice structure geometry with a diamond pore size of 700 µm and a porosity of 80% [12].

There is a lack of literature focusing on the outcomes of titanium cages in ACDF, and it is vital that the literature continuously evolves to include more surgical data. This retrospective study aims to evaluate the early radiological outcomes of the CONDUIT^TM^ EIT Cellular Titanium Cervical Cages in ACDF procedures in Northern Ireland, both with and without anterior plating. This aim includes assessing the implant’s early safety and efficacy; and to provide initial data to contribute to the existing literature and guidelines.

## Materials and methods

Patient cohort

All patients who underwent ACDF with a CONDUIT^TM^ EIT Cellular Titanium Cervical Cage in the BHSCT were identified following contact with a representative from the implant company, with this being the inclusion criterion for this study. A total of four spinal surgeons operated across all cases. All patient information was documented using the Northern Ireland Electronic Care Record (NIECR). Demographic data collected included age and sex; indication for surgery and the American Society of Anaesthesiologists (ASA) grade were also recorded. Surgical information recorded included the level of operation, the performing surgeon, if anterior plating was required, and if there was a return to surgery. Decisions on anterior plating were made following spinal multidisciplinary team meetings amongst spinal orthopaedic consultants within the BHSCT based on common factors like level of injury, vertebral column involvement, patient co-morbidities, and severity of injury. Post-operative and intra-operative complications were documented, and admission and discharge dates were noted to calculate the length of hospital stay. By maintaining patient anonymity in accordance with the Data Protection Act of 1998, ethical approval was not required for this retrospective study.

Surgical approach

**Figure 1:**
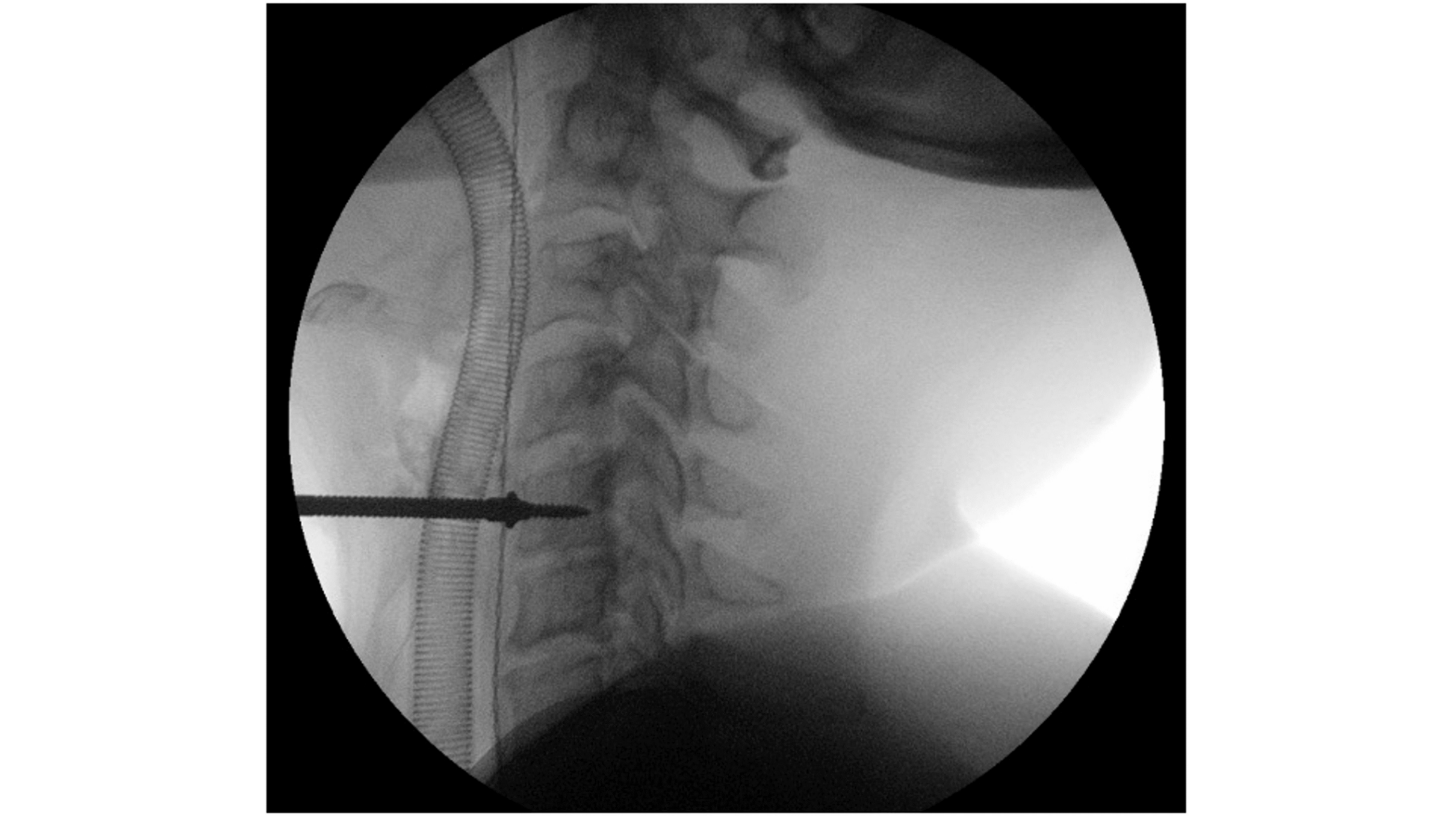
Intraoperative fluoroscopy image of a distracting Caspar pin in the C5 vertebral body during a C5/C6 anterior cervical discectomy and fusion procedure

In this study, all ACDF procedures were performed using the Smith-Robinson approach. A Mayfield clamp was applied, and the patient was positioned supine with a jelly roll between the shoulders. Standard preparation and draping for sterility were undertaken. A fluoroscopic-guided image was taken to confirm the level of surgery. Horizontal skin crease incision in line with Langers lines was made, and blunt dissection utilized to approach the cervical spine. Vertebral body Casper pin distraction was utilised prior to performing the discectomy (Figure 1). The discectomy was performed using Kerrison rongeurs and pituitary forceps. The posterior longitudinal ligament and any posterior osteophytes were excised to visualise the dura and nerve roots in the foramen. The vertebral body endplates were prepared with the use of a curette. The final implant size was determined using a trial template, with the aim of approximating the original pre-degenerative disc height. Intraoperative fluoroscopy imaging was used to determine satisfactory cage positioning. Satisfactory haemostasis and lavage were obtained prior to skin closure.

Radiological analysis

Radiographic images were analysed using the Northern Ireland Picture Archiving and Communication System (NIPACS) Sectra Software. Patients were assessed radiologically at three time points: pre-operatively, day one post-operatively, and at follow-up, an average of 6.19 months later. Lateral cervical plain film X-rays were assessed, and where they were not available, whole spine Magnetic Resonance Imaging (MRI) was used. All measurements were recorded at each time point with the patient in a neutral anatomical position. 

Intervertebral disc height

**Figure 2:**
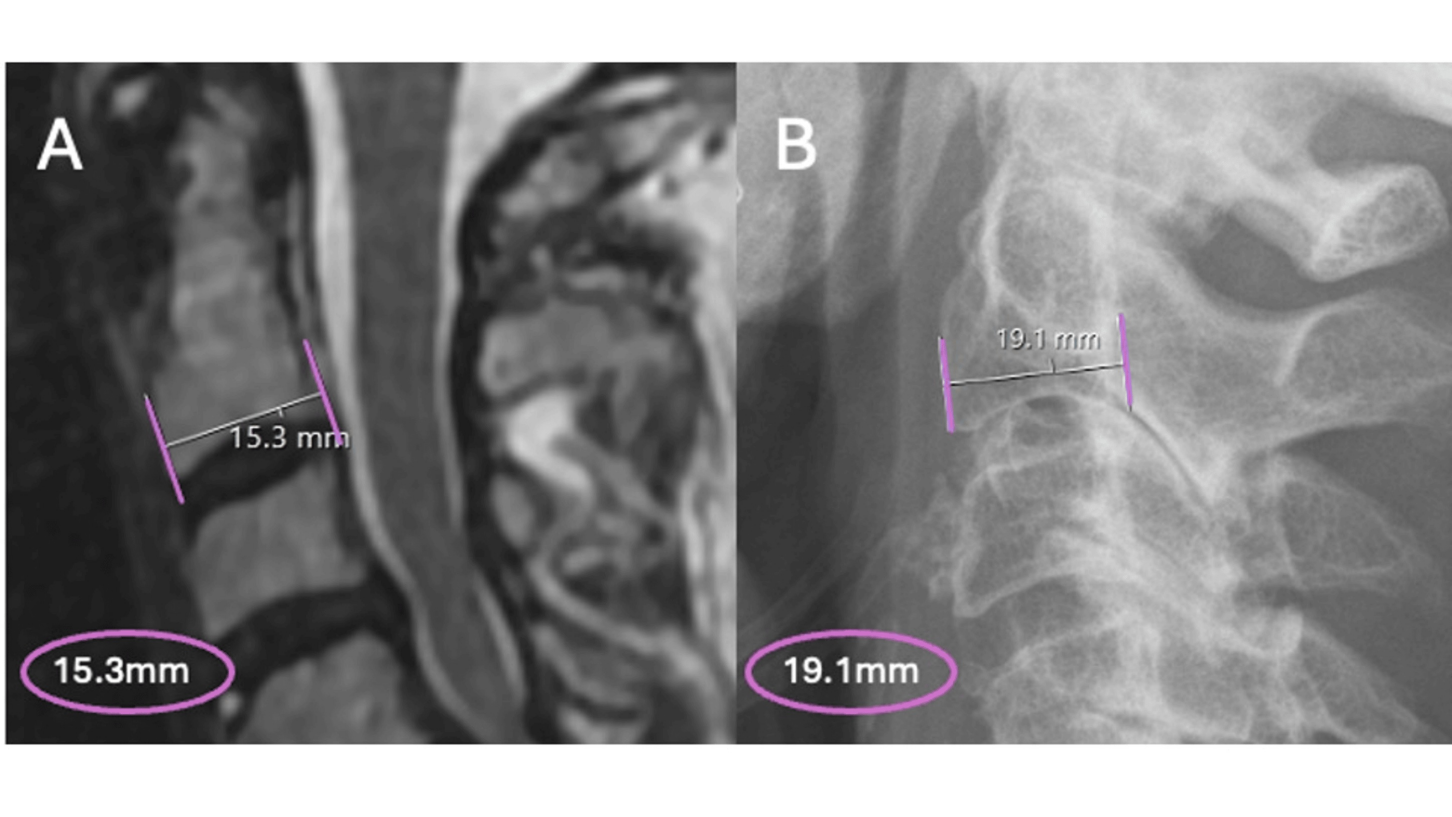
Anteroposterior measurement of the second cervical vertebrae on MRI versus X-ray of the same patient to demonstrate calibration difference A. MRI showing AP C2 distance as 15.3 mm B. Lateral cervical X-ray of the same patient showing AP C2 measurement as 19.1 mm. This highlights the importance of calculating the calibration difference to prevent errors in measurements. MRI: magnetic resonance imaging; AP: anteroposterior.

Intervertebral disc height (IDH) of the operated segment was recorded, and the percentage change was calculated between each of the three time points. The distance between two adjacent vertebral bodies at the midpoint of each superior and inferior endplate was used to calculate the IDH. Due to the magnification error between X-ray and MRI, calibration was required in patients who had the intervertebral disc height measured on X-ray (DH) to avoid discrepancy and accurately calculate the actual disc height (aDH) as equivalent to MRI measurements. Following the study conducted by Shigematsu et al. [13], the anteroposterior length of the C2 vertebral body on X-ray (AP) was used as a reference point (Figure 2) calibrated against the same measurement on MRI (aAP). McKee et al. proposed the formula aDH = aAP/AP x DH, which we used to calculate the actual disc height [14].

Segmental Cobb angle

The degree of kyphosis/ lordosis of the operated segment was measured using the segmental Cobb angle. Using the NIPACS angle function, two lines are drawn across the superior vertebral endplate of the superior vertebral body of the affected segment, and another line across the inferior vertebral endplate of the inferior vertebral body. PACS will simulate a perpendicular line drawn from each parallel line, and the angle where they intersect is the segmental Cobb angle. Lordosis is a positive value, and kyphosis is a negative value in this study. Calibration was not required for the lateral angle measurements on MRI and X-ray.

Overall Cobb angle

**Figure 3:**
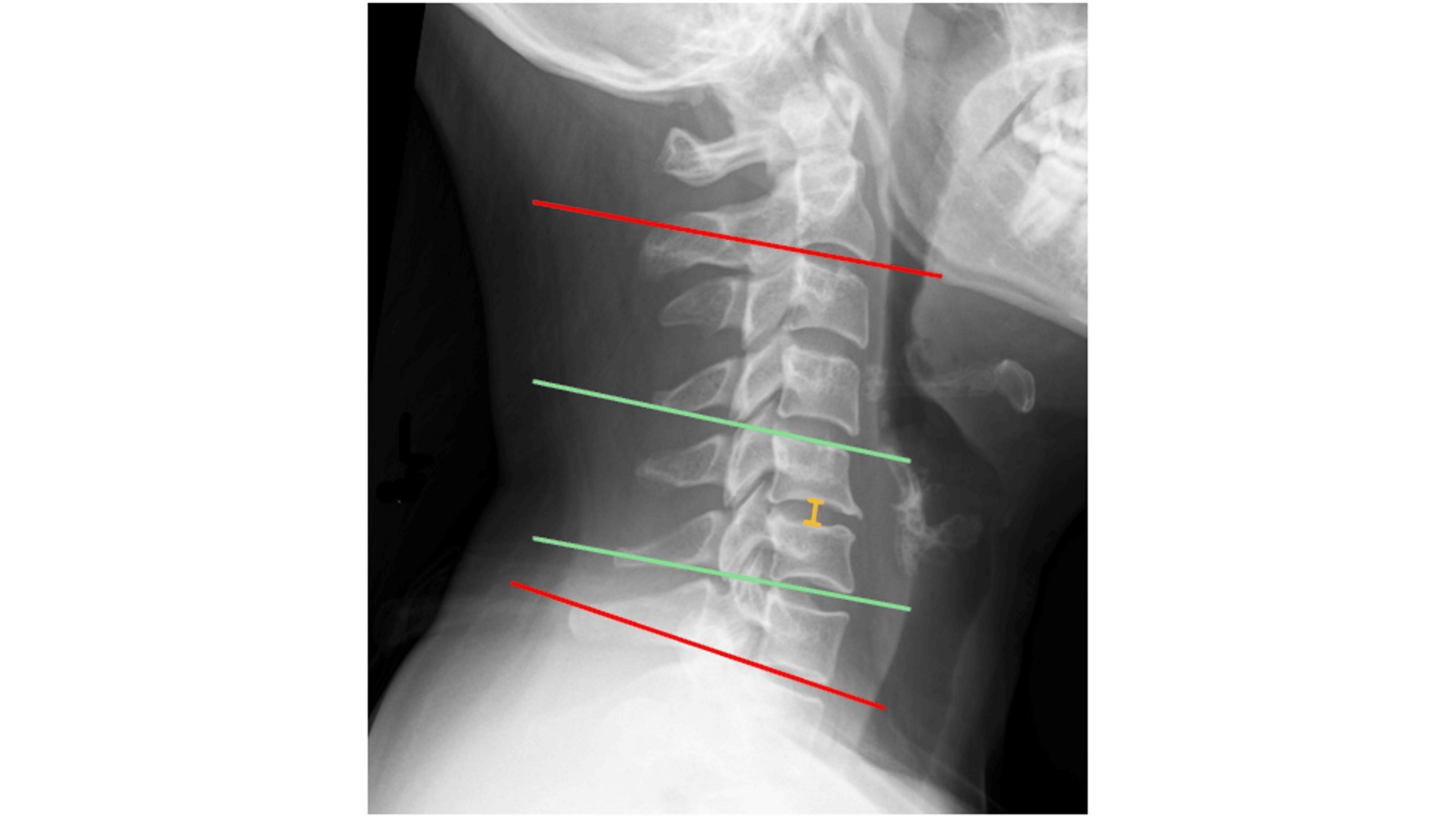
Radiological outcomes measured Pre-operative lateral cervical X-ray of a patient with C5/C6 disc degeneration and loss of lordosis of the cervical spine. Red: Overall Cobb Angle, Green: Segmental Cobb Angle, Yellow: Intervertebral Disc Height.

The degree of lordosis/kyphosis of the whole cervical spine was measured according to the overall C2-C7 Cobb angle (Figure 3). Using the NIPACS angle function, two lines are drawn across the inferior vertebral endplates of C2 and C7. PACS will simulate a perpendicular line drawn from each parallel line, and the angle where they intersect is the overall Cobb angle.

Cage movement

Cage migration was assessed between the day one post-operative and follow-up X-rays. If established, the distance between the posterior aspect of the vertebral body and the posterior aspect of the cage was measured to establish the degree of migration. Where cage subsidence was obviously visible on X-ray, the total intervertebral height of the operated segment from the midpoint of the superior endplate of the cranial vertebrae to the midpoint of the inferior endplate of the caudal vertebra was measured as described by Ha et al. [15]. This was measured post-operatively and at follow-up to establish any decrease in height. Ha et al. described subsidence as a total intervertebral height loss of >3 mm [15]. Using an adjusted version of the McKee et al. formula, the magnification error from MRI to x-ray was remedied [14]. Any clearly identifiable post-operative fusion demonstrated on X-ray was recorded. 

Inter-observer reliability

All measurements were performed on digital radiographs using NIPACS software to measure angles and distances accurately to 0.1 degrees and 0.01 mm, respectively. To validate the assessed data, all images were reviewed independently by the author and an independent observer (Spinal Physician Associate in the Trauma and Orthopaedic Department of the BHSCT). Inter-observer reliability was determined for all three radiographic measurement types (IDH, segmental Cobb angle, and overall C2-C7 Cobb angle) to obtain the intraclass correlation coefficient (ICC) for each measurement. This method was used as all data contained continuous numerical values. Following the guidelines proposed by Koo et al. [16], an ICC >0.90 indicated excellent reliability, 0.75-0.90 being good, 0.5-0.75 being moderate, and <0.5 being poor. 

Statistical analysis

Statistical analysis was performed using Statistical Package for Social Sciences (SPSS) software, version 29.0 (IBM Corp., Armonk, NY), with statistical significance set at a p-value <0.05. All values were expressed as the mean ± standard deviation. To evaluate the significance of changes in each radiological outcome between the three time points, the Wilcoxon signed rank test was employed. Specifically, the test was applied to the differences in radiological outcomes between pre-operative and post-operative, post-operative and follow-up, and pre-operative and follow-up for all three measurements: intervertebral disc height, segmental Cobb angle, and overall C2-C7 Cobb angle. A Spearman's rank correlation (SRC) analysis was conducted to examine the overall relationship between all three radiological variables from the pre-operative period to the follow-up period. Linear graphs were also produced using the SPSS software.

## Results

Patient cohort

**Figure 4:**
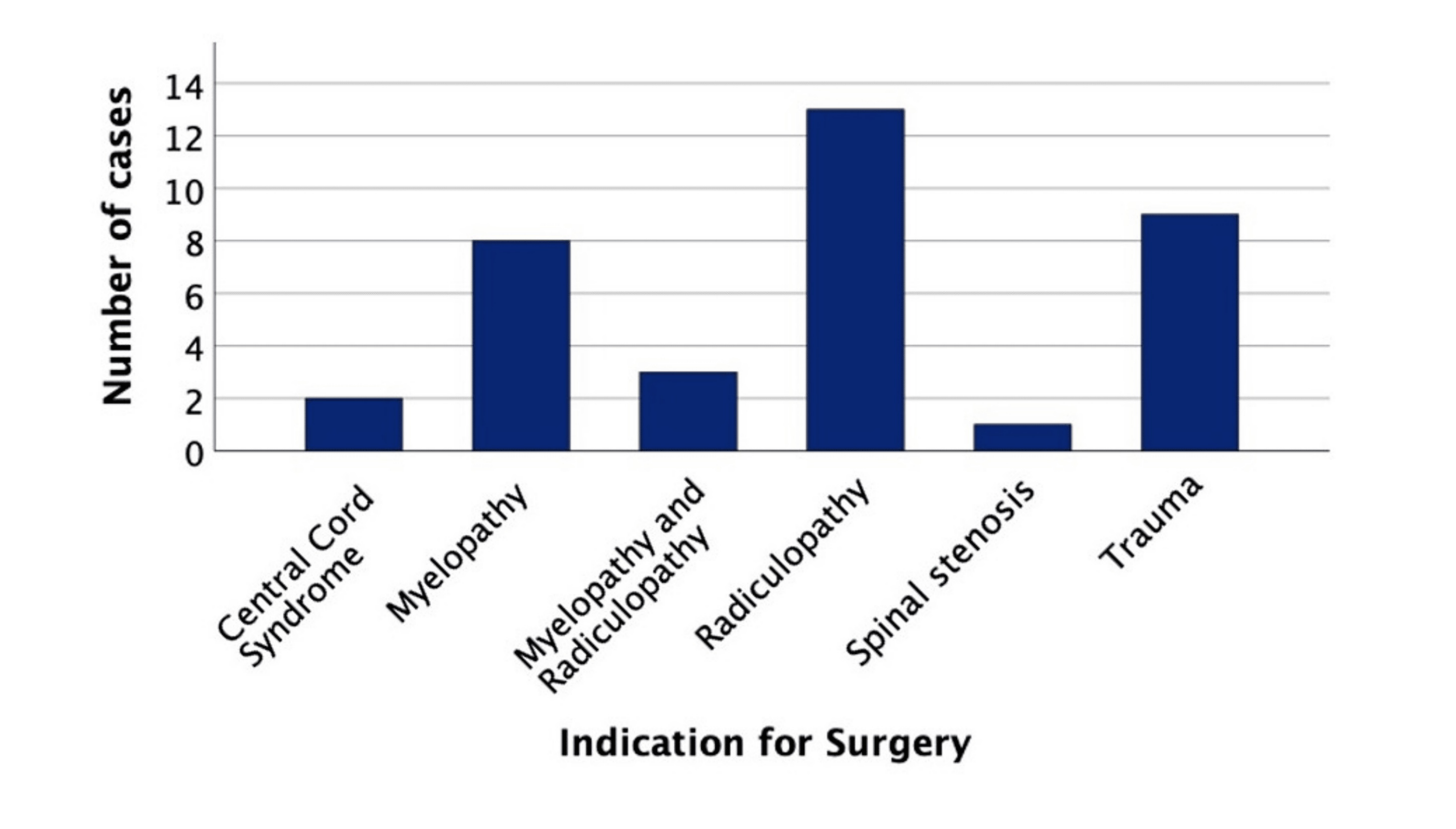
Bar chart displaying the indication for surgery in the patient cohort

From May 2022 to December 2023, 29 patients underwent a total of 36 ACDF procedures with the CONDUIT^TM^ EIT Cellular Titanium Cage. Among the cases, 23 were male, while the remaining 13 were female (Table 1). The mean age was 57 ± 13.9 and most patients had an ASA of one/two (80.55%). Radiculopathy was the most common indication for ACDF surgery in 13 of the cases with trauma being the second most common in nine cases (Figure 4). 

**Table 1:** Patient demographics and surgical information CONDUIT^TM  ^(AO Foundation, Davos, Switzerland)

	CONDUIT^TM^ Cage Patients
Total number of cases	36
Sex (Male: Female)	63.89% : 36.11%
ASA grade	
1	8.33%
2	72.22%
3	16.67%
4	2.78%
Patients with anterior plating	16.67%
Surgeon	
A	50%
B	27.78%
C	13.89%
D	8.33%
Mean follow-up (months)	6.19

**Figure 5:**
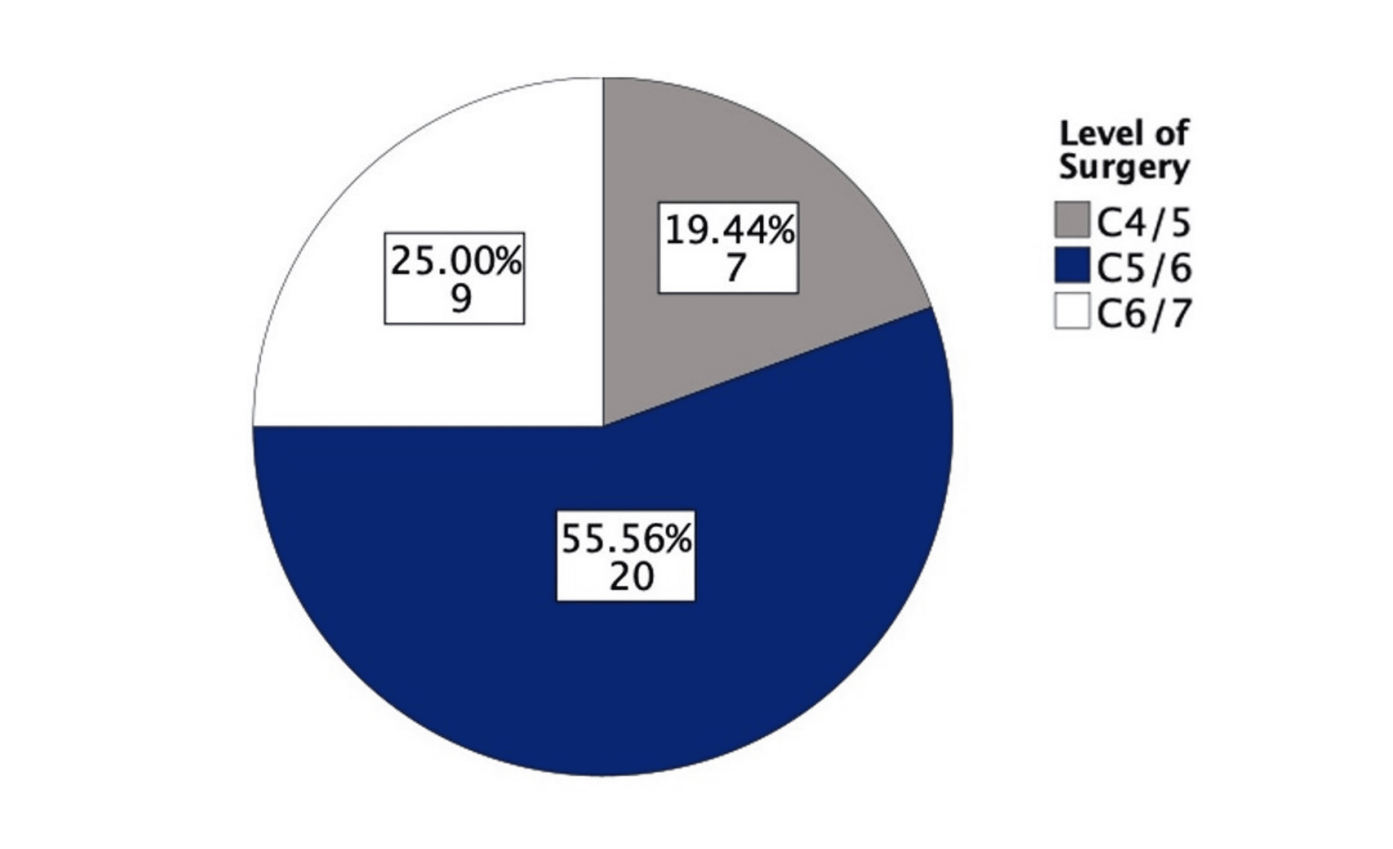
Pie chart displaying the vertebral levels operated upon

Six cases required anterior plating of the vertebral bodies, with five of these being trauma cases. The most common vertebral level operated on was C5/C6 in 20 (55.56%) cases, followed by C6/C7 in nine (25%) cases (Figure 5). The average length of hospital stay (LOS) across all cases was four days, with stays ranging from one to 26 days, and the most common duration being one day. When excluding trauma and central cord syndrome cases, the average LOS decreased to 2.85 days. 

Surgical approach and complications

There were no technical complications during surgery, and no patients were required to return to surgery. Four (11.1%) procedures experienced mild dysphagia post-operatively, with half of these patients having received anterior plating due to trauma. All four patients were discharged from the Speech and Language Therapy service upon their discharge from the hospital and reported resolution of symptoms at their next follow-up appointment, an average of 9.57 months later.

Intervertebral disc height

**Figure 6:**
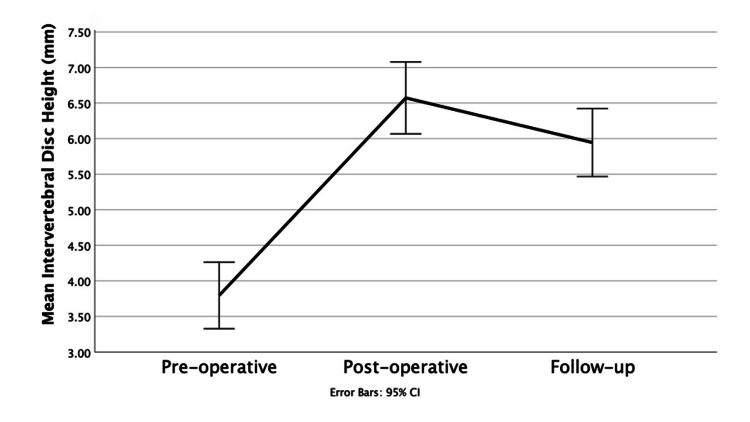
Intervertebral disc heights at different time points Line graph showing intervertebral disc height (mm) at the three time points: pre-operative, post-operative and follow-up.

The mean ICC for all IDH measurements was 0.95, indicating excellent inter-observer reliability. The IDH showed a statistically significant immediate increase from pre-operative imaging to post-operative imaging with an average increase of 2.77 ± 1.95mm (p<0.001). From the post-operative imaging to the follow-up period, there was a significant decrease in IDH of an average of 0.62 ± 0.98mm (p<0.001). The results are displayed in Figure 6 as a line graph and in Table 2.

**Table 2:** Average IDH measurements and mean changes at three time points with statistical significance IDH: Intervertebral disc height; Pre: pre-operative; Post: post-operative; Follow: follow-up. *Statistical significance at p <0.05.

Measurement	Mean ± SD (mm)	Wilcoxon Signed Rank (p-value)
Average IDH		
Pre	3.79 ± 1.38	-
Post	6.57 ± 1.49	-
Follow	5.94 ± 1.41	-
Mean change in IDH		
Pre to Post	+ 2.77 ± 1.95	<0.001*
Post to Follow	-0.62 ± 0.98	<0.001*
Pre to Follow	+ 2.14 ± 1.98	<0.001*

**Figure 7:**
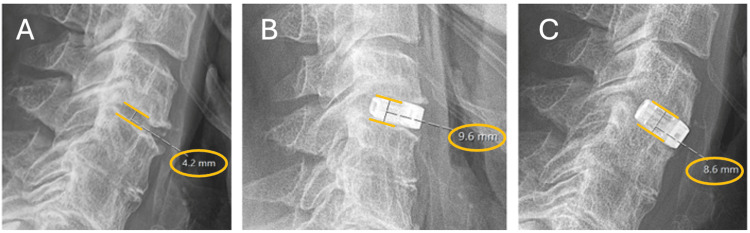
Change in Intervertebral disc height of the C4/C5 disc space A. Pre-operative disc height of 4.2mm prior to ACDF surgery B. Immediately post-operative the disc height has increased to 9.6mm C. Slight decline of the disc height to 8.6mm at follow-up.

Overall, from the pre-operative imaging to the follow-up imaging, there was still a significant increase in IDH of an average 2.14 ± 1.98 mm (p<0.001) despite a decline from post-operative to follow-up period. An example of the overall trend is shown by a patient case in Figure 7. A total of five cases had an overall decrease in IDH from pre-operative to follow-up of an average of 1.4mm, with three of these being trauma cases. 

Cage movement

**Figure 8:**
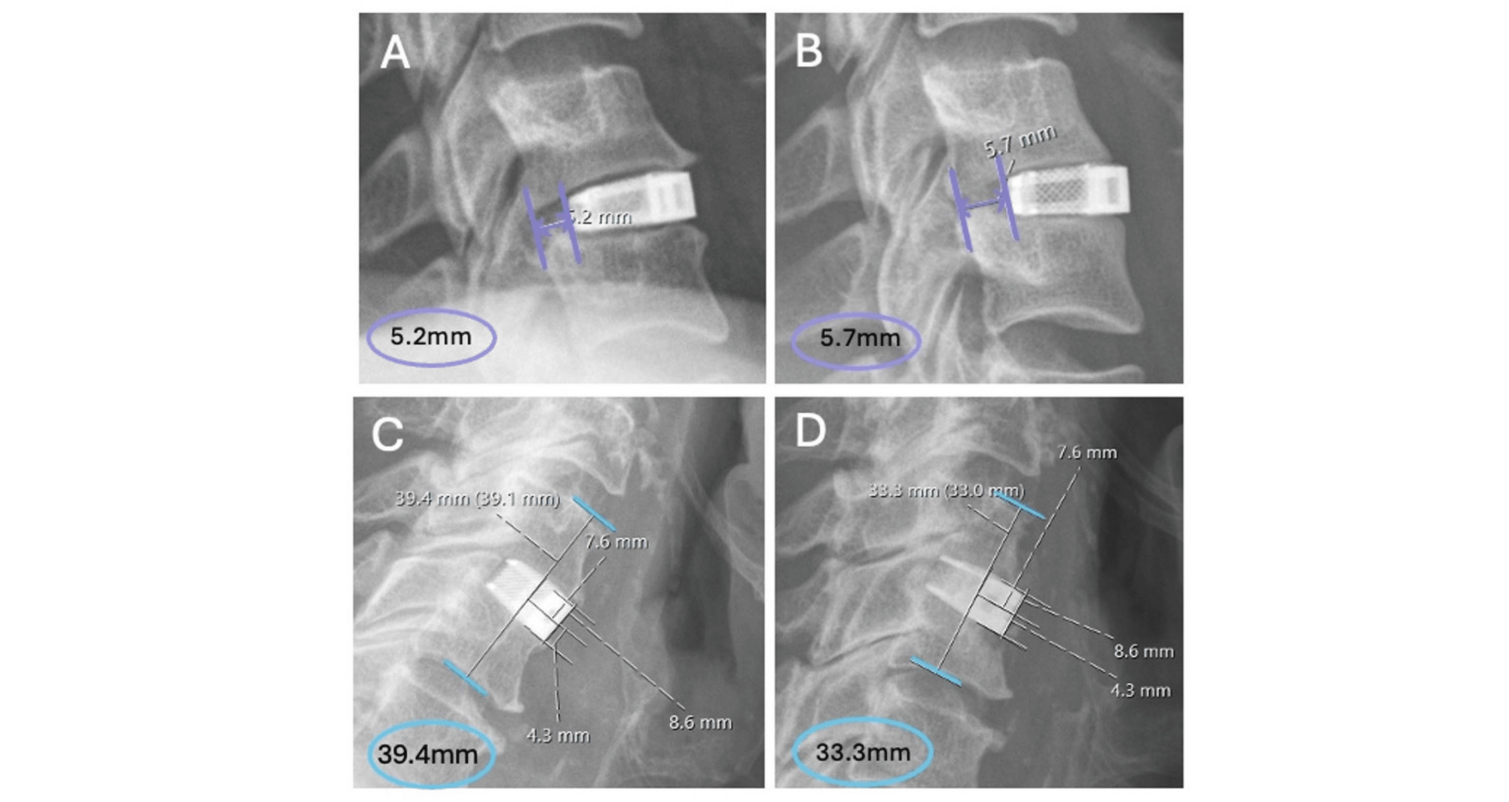
Anterior cage migration examples Examples of anterior cage migration between post-operative (A) and follow-up (B) time points and cage subsidence between post-operative (C) and follow-up (D) time points.

**Figure 9:**
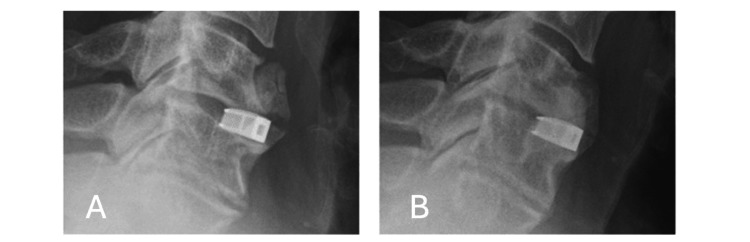
A case showing ideal vertebral body fusion A. Day one post-operative x-ray showing the implant in the intervertebral space B. Follow-up x-ray showing vertebral body fusion and osteointegration of the titanium implant.

Cage subsidence was noted in one case where the vertebral segment height on the immediate post-operative X-ray was 39.4 mm, whilst the most recent X-ray, 3.45 months after surgery, showed the vertebral segment height as 33.3 mm (Figure 8), indicating a decline in height. One case of anterior cage migration was observed where, upon initial post-operative X-ray, the posterior aspect of the cage was 5.2mm anterior to the back of the vertebral body, and upon the follow-up X-ray 3.53 months later, the same distance was measured as 5.7 mm (Figure 8). Both cases were only discovered through radiological review, and both patients were asymptomatic. There was one obvious case of vertebral body fusion noted (Figure 9). This patient’s follow-up x-ray was 11.5 months after the ACDF surgery.

Segmental Cobb angle

**Figure 10:**
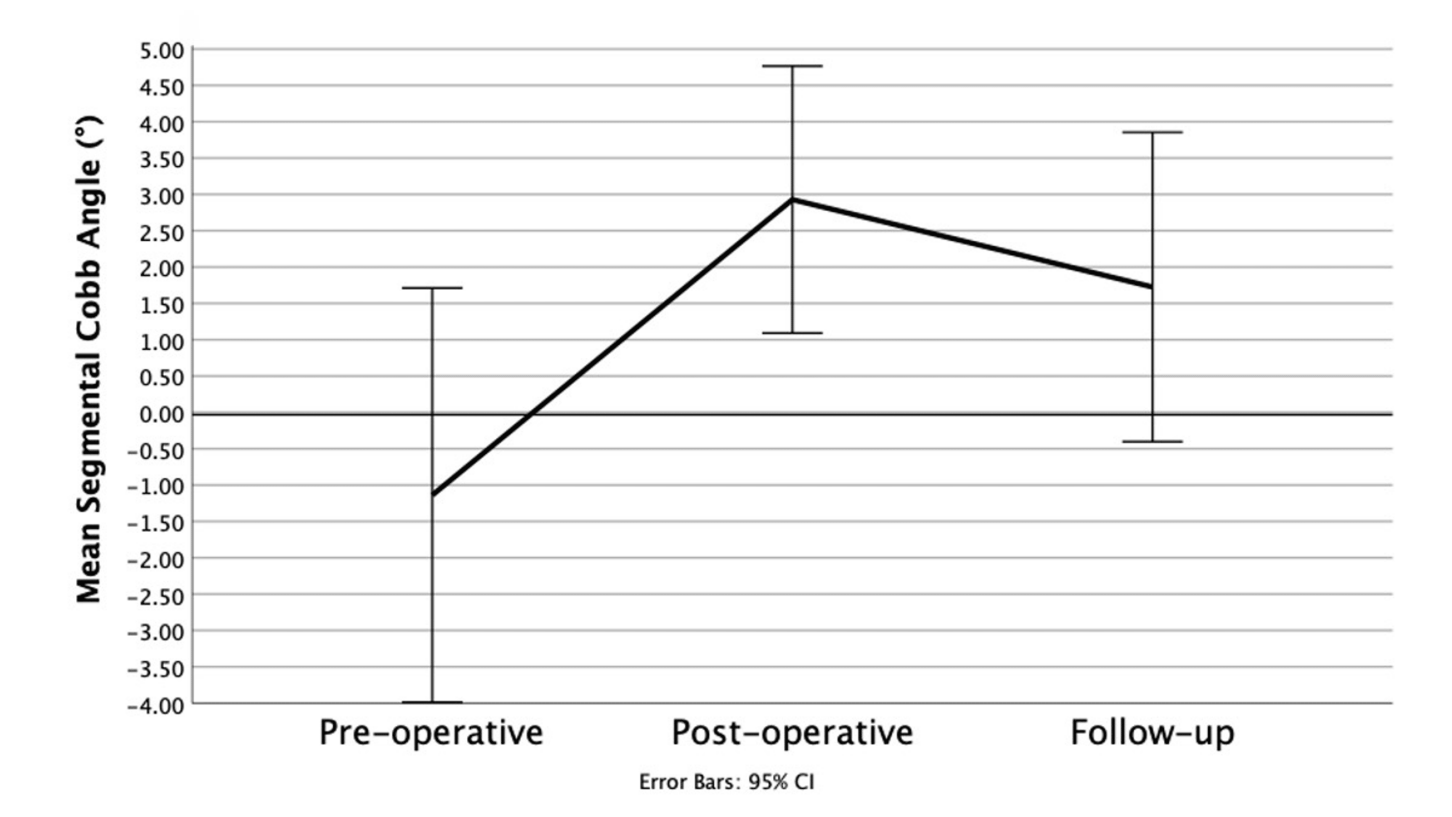
Segmental Cobb angle at the three time points Line graph showing segmental Cobb angle at the three time points: pre-operative, post-operative and follow-up.

**Figure 11:**
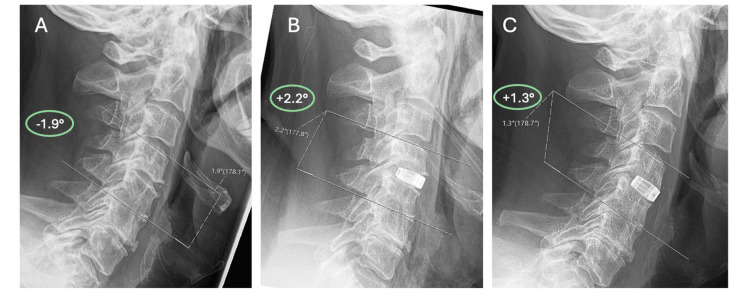
Change in segmental Cobb angle A. Pre-operative angle measuring -1.9° showing kyphosis B. Immediately post-operative the angle has increased to a lordotic value of 2.2° C. Follow-up angle of 1.3° showing a slight loss of lordosis from post-operative imaging, but still overall segmental restoration of lordosis.

The ICC for segmental Cobb angle is 0.92, showing excellent inter-observer reliability. The segmental Cobb angle showed immediate restoration of cervical lordosis from initial kyphosis from the pre-operative period to the post-operative period with an average increase of +4.08 ± 7.60° (p=0.003). There was a slight loss of lordosis from the post-operative period to the follow-up period, with a loss of 0.96 ± 1.39° (p=0.007) of the segmental Cobb angle. Although the change is small, it is statistically significant. An example of the overall trend is shown by a patient case in Figure 11. However, lordosis was still maintained at the follow-up period with an average segmental angle of 1.71 ± 6.28°. Overall, there was restoration of segmental lordosis with the segmental Cobb angle increasing from the pre-operative period to the follow-up period by an average of +2.86 ± 7.60° (p=0.045), showing a significant change from kyphosis to lordosis. The results are displayed in a line graph in Figure 10 and in Table 3.

**Table 3:** Average segmental Cobb angle and mean changes at three time points with statistical significance Pre: pre-operative; Post: post-operative; Follow: follow-up. *Statistical significance at p <0.05.

Measurement	Mean ± SD (°)	Wilcoxon Signed Rank (p-value)
Mean Segmental Cobb Angle (°)		
Pre	-1.14 ± 8.42	-
Post	2.93 ± 5.43	-
Follow	1.71 ± 6.28	-
Mean change in Segmental Cobb Angle (°)		
Pre to Post	+4.08 ± 7.60	0.003*
Post to Follow	-0.96 ± 1.39	0.007*
Pre to Follow	+2.86 ± 7.60	0.045*

Overall C2-C7 Cobb angle

**Figure 12:**
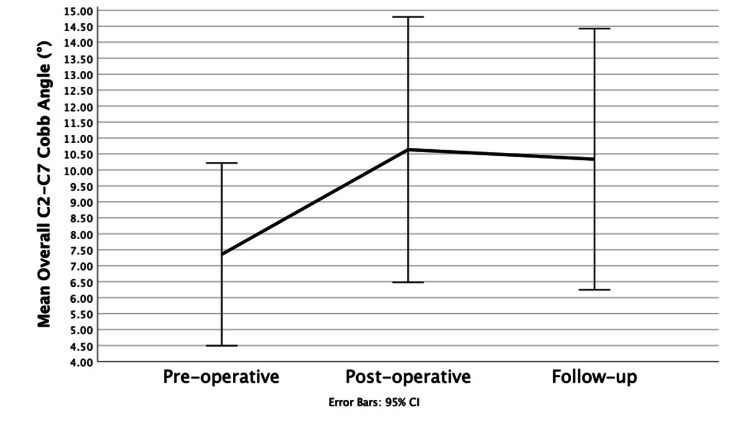
Overall C2-C7 Cobb angle at the three time points Line graph showing overall C2-C7 Cobb angle at the three time points: pre-operative, post-operative and follow-up.

**Figure 13:**
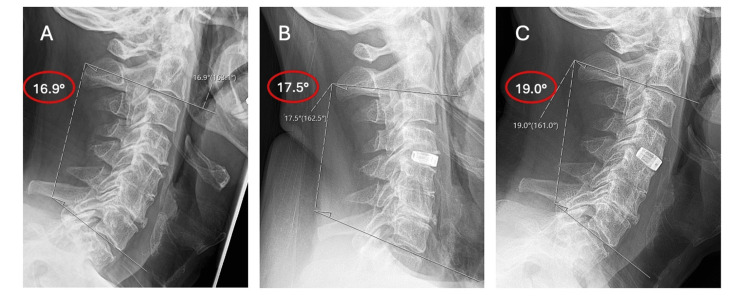
Change in overall C2-C7 Cobb angle A. Pre-operative angle of 16.9° showing lordosis B. Immediately post-operative angle of 17.5° showing increased lordosis C. Follow-up angle of 19.0° showing further lordotic change over the follow-up period.

The ICC for the overall C2-C7 Cobb angle is 0.88, indicating good inter-observer reliability. The overall C2-C7 Cobb angle also showed immediate improvement in cervical lordosis from the pre-operative period to the post-operative period with an average increase of 3.28 ± 11.14° (p=0.026). Similar to the segmental Cobb angle, there is also a slight loss of lordosis noted from the post-operative period to the follow-up period, with an average loss of 0.29 ± 8.46° (p=0.422) of the overall Cobb angle. An example of the change in a patient’s overall Cobb angle is shown in Figure 13. The overall Cobb angle still showed an improvement in lordosis from pre-operative to follow-up, with an overall increase of 2.98 ± 9.94° (p=0.021) from a pre-operative average value of 7.36 ± 8.46° to a follow-up average of 10.64 ± 12.29°. The results are displayed in a line graph in Figure 12 and in Table 4.

**Table 4:** Average overall C2-C7 Cobb angle and mean changes at three time points with statistical significance Pre: pre-operative; Post: post-operative; Follow: follow-up. *Statistical significance at p <0.05.

Measurement	Mean ± SD (°)	Wilcoxon Signed Rank (p-value)
Mean Overall Cobb Angle (°)		
Pre	7.36 ± 8.46	-
Post	10.64 ± 12.29	-
Follow	10.34 ± 12.09	-
Mean change in Overall Cobb Angle (°)		
Pre to Post	+3.28 ± 11.14	0.026*
Post to Follow	-0.29 ± 8.46	0.422
Pre to Follow	+2.98 ± 9.94	0.021*

There was a statistically significant moderate positive SRC of 0.498 (p=0.002) between the change in IDH and the change in overall C2-C7 Cobb angle. This suggests that an increase in IDH is associated with an increase in the overall Cobb angle. Additionally, the SRC between change in IDH and change in segmental Cobb angle was 0.238 (p=0.162), indicating a weak positive relationship, although not statistically significant. Finally, the SRC between overall Cobb angle and segmental Cobb angle was -0.17 (p=0.92), indicating a very weak negative relationship, which was also not statistically significant. Therefore, we cannot conclude a meaningful association between these two variables.

## Discussion

Patient cohort

The most operated upon level in this study was C5/6 (55.56%), and this is in keeping with the fact that most of the flexion and extension action of the cervical spine occurs between C4-C6, with C5/6 associated with the greatest range of movement, followed by C6/C7 [17]. In the current study, 16.7% of patients had anterior plating; most of these patients (83%) were trauma cases. This study also found that the overall rate of dysphagia was 11.1% and identified the link between dysphagia and anterior plating, with 50% of patients with dysphagia receiving anterior plating due to trauma. In unstable cervical spinal injuries, anterior plating can provide stabilization and support for the spine [18] as it acts to bear part of the spinal load [9]. Anterior plating is widely considered not to produce inferior neurological outcomes [18]. However, it increases post-operative dysphagia rates and increases the operation time, which can lead to increased complications [9]. 

The average LOS for all 29 patients was 3.89 days. This ranged from one to 26 days, with the mode being one day, showing a fast post-operative recovery. Arnold et al. found the average LOS following ACDF was 1.98 (± 1.6) days; however, their retrospective study of 103 patients only included elective procedures, therefore not considering how trauma can increase post-operative LOS [19]. After excluding trauma cases from this study, the average LOS decreased to 2.85 days. This aligns closely with Lee et al.'s multicentre study of 182 patients, which found that 56.6% of patients undergoing elective ACDF procedures had a LOS of less than two days [20].

Intervertebral disc height

In this study, the mean IDH increased on average by 78.43% from the pre-operative imaging to the follow-up imaging, with a significant average increase of 2.14 ± 1.98mm (p<0.001). This showed that the EIT Cellular Titanium Cage is successful in increasing overall IDH. From pre-operative to post-operative imaging, the IDH increased from an average of 3.79 ± 1.38 mm to 6.57 ± 1.49 mm. There was a decrease from the post-operative period to the follow-up by -0.62 ± 0.98 mm with an average follow-up value of 5.94 ± 1.41 mm. This pattern of IDH change is reproduced in similar studies by Abudouaini et al. [21], Deng et al. [22], and McKee et al. [14], with all three experiencing an immediate post-operative rise in IDH followed by a gradual loss over the follow-up period. This may be explained by localised pressure on the cage leading to a decrease in IDH [10]. Deng et al. [22] followed up six months post-operatively, whereas Abudouaini et al. [21] followed up to one year and therefore showed more loss in IDH due to the longer period; however, both still demonstrated an overall increase in IDH from pre-operative imaging to follow-up. 

Deng et al. [22] patient cohort underwent 26 ACDF procedures with titanium cages for degenerative disease, followed up six months post-operatively, mirroring conditions very similar to this study. Notably, they also included another measurement of the IDH at three months post-operative, providing more specific information on loss of IDH and its associated timing over the follow-up period. Slightly more IDH (0.4 mm) was lost between one week post-operative and three months post-operative, and 0.3 mm was lost between three months post-operative and six months post-operative, showing that more IDH decline happened in the immediate follow-up period. This could be attributed to vertebral body settling post-procedure. Whilst their study is very similar to the current study, a limitation that reduces the comparability is the difference in the method of measuring the IDH. Deng et al. measured the anterior and posterior intervertebral height between the vertebral bodies and then calculated the average to determine the IDH [22]. This contrasts with the current study’s methodology, which may introduce slight variances in the data collected. However, despite these methodological differences, the results showed very similar values and trends. Both studies indicate that the most significant decline in IDH occurs shortly after the procedure, suggesting a consistent pattern of vertebral body settling.

Abudouaini et al. assessed zero-profile (ZP) implants in their study, meaning that although direct comparisons cannot be drawn between ZP and titanium plates regarding IDH, it shows that titanium cages in ACDF procedures are similar to those of other types of implants; thereby helping to establish their non-inferiority [21]. The method for measuring IDH in the Abudouaini et al. [21] study was the same as the current study, reinforcing that the results can be accurately compared and validating the trend seen in IDH. Abudouaini et al. [21] concluded that a post-operative loss in disc height had no relation to symptoms over the follow-up period.

The slight loss in IDH from the post-operative time point to follow-up is thought to be osteointegration of the porous titanium cage. The EIT Cellular Titanium Cage shows complete osteointegration histologically at two years post-operative [23], so it could be theorised that the decline in IDH might continue until then. Although this can be implied, the study from van den Brink et al. [23] involved explanting and assessing the bone growth histologically within the implant, therefore this is unable to be reproduced or concluded in this study. While the results from their study are valuable, their limitation lies in being based on a single case example.

Patients who have a post-operative IDH of 6-8 mm have a better cervical range of motion than those patients with an IDH of less than 6 mm post-operatively [24]. Furthermore, Li et al.'s study of 160 patients undergoing ACDF procedures showed that patients with a pre-operative IDH of less than 4 mm had worse degenerative disease, leading to greater disc collapse, and hence these patients benefited more from ACDF in improving cervical ROM and flexion and extension [24]. The study measured flexion and extension by lateral flexed and extended cervical X-rays, which they considered an effective way to measure functional outcomes. Although the current study did not measure ROM, incorporating it into future studies would provide insight into its relation to disc height and post-operative functional outcomes. Although this is a potential limitation for this study, lateral cervical flexion and extension X-rays are not performed in the BHSCT so as not to expose patients to excess radiation when the results would not change the existing clinical management. This highlights the importance of balancing comprehensive data collection with patient safety, suggesting future studies should explore alternative, nonradiative methods for assessing ROM to address this gap.

Segmental Cobb angle

The segmental Cobb angle demonstrated restoration of cervical lordosis to 2.93 ± 5.43° from a pre-operative kyphotic average of -1.14 ± 8.42°. From the post-operative imaging to follow-up there was a reduction in lordosis from 2.93 ± 5.43° to 1.71 ± 6.28°. Despite the slight loss of lordosis in the follow-up period, the implant restores overall lordosis to the affected segment.

Hosoi et al. conducted a similar study with 36 patients undergoing single-level ACDF with titanium cages for degenerative cervical spine disease [25]. They found results consistent with the pattern in the current study, and both studies showed a statistically significant increase in segmental Cobb angle from the pre-operative to follow-up period. In the Hosoi et al. study, the segmental cob angle began kyphotic at a baseline of -1.4 ± 4.3° and showed post-operative restoration of lordosis to 6.3 ± 4.9° [25]. However, they observed a greater reduction in lordosis from post-operative imaging to follow-up, averaging 0.4 ± 6.0° at follow-up. This could be explained by a follow-up period spanning a minimum of one year to 96 months, leading to a much longer observational period compared to the current study. The extended follow-up in the Hosoi et al. [25] study offers a more comprehensive view of the long-term sustainability of titanium cages and their long-term progression in ACDF, which the current study, with its shorter follow-up, could not capture. Longer follow-up periods can be influenced by external factors not accounted for in the study design. On the other hand, the current study’s shorter follow-up period allows for a more immediate assessment of post-operative radiological outcomes, ensuring that the findings are relevant to the early stages of patient recovery following ACDF surgery.

Hosoi et al.’s (ibid) study has the same patient cohort size as the current study and uses the same imaging modality (lateral cervical X-rays) at the same timepoints: pre-operative and immediately post-operative. The only difference is the much larger follow-up period. The validity of the Hosoi et al. [25] study is strengthened by its consistency with the current study in terms of patient cohort size, methodology, and use of titanium implants, allowing for accurate comparison and reliable findings. Both studies have limitations due to their small sample sizes, which may affect the validity of the results. Moreover, while the statistical methods used in both studies were appropriate for the data, the small sample sizes raise concerns about the studies' ability to detect subtle but clinically significant differences. Future studies should aim for larger, randomized cohorts.

Katsuura et al. also associated adjacent intervertebral disc degeneration with segmental post-operative kyphotic malalignment after ACDF, highlighting the need for segmental lordotic restoration [26]. Furthermore, Hu et al. established that achieving cervical spine lordosis is associated with an improvement in the Neck Disability Index (NDI) score and clinical outcomes as opposed to patients who retained kyphotic measurements post-operatively [27]. Their study categorised patients into three groups: patients who were lordotic pre-operative and maintained it, patients who were kyphotic pre-operative and became lordotic post-operative, and patients who stayed kyphotic from pre-operative to post-operative. The main recognised limitation of the Hu et al. study is the disproportionate group sizes, with 64 patients maintaining lordosis from pre-operative to post-operative, compared to only 23 in the kyphotic group and 17 in the restored group [27]. This imbalance could exaggerate the outcomes due to the larger sample size of the maintained lordosis group, potentially skewing the results towards more favourable outcomes for this alignment. The authors themselves acknowledged this limitation and suggested a prospective matched controlled outcomes study to address it.

When comparing titanium cages to other common cages, such as PEEK cages, it is suggested that there is no significant difference in post-operative loss of segmental lordosis or clinical outcomes, but that titanium cages led to increased numbers of cage subsidence [10,28]. 

Overall C2-C7 Cobb angle

C2-C7 Cobb angle was 7.36 ± 8.46° pre-operatively, significantly improved to 10.64 ± 12.29° post-operatively (p=0.026) and showed a decrease (p=0.422) to 10.34± 12.09° at follow-up. Overall improvement of Cobb angle from pre-operative imaging to follow-up was significant at 2.98 ± 9.94° (p=0.021).

Chen et al. compared titanium and PEEK cages in multilevel ACDF procedures [11]. Both groups had a similar number of patients with titanium, having 29, and PEEK, having 30, allowing for minimal bias between the groups. In the titanium group, there was an average pre-operative measurement of 6.83 ± 8.83°, which increased significantly (p<0.05) to 16.49 ± 10.27° post-operatively and then declined to 7.86 ± 8.52°. Chen et al. found that the overall Cobb angle in the PEEK cages group also increased significantly from pre-operative to post-operative and that between the two cages, there was no statistical significance in terms of post-operative outcomes (p>0.05) highlighting the non-inferior nature of the titanium implant in improving post-operative disc height [11]. At the follow-up (86-116 months later) the titanium group was 7.86 ± 8.52°, having had an overall loss of correction of 8.59 ± 4.67° compared to this study’s 0.29 ± 8.46° loss from post-operative to follow-up. Again, they had a significantly longer follow-up period ranging from 86-116 months, compared to the current study’s average of 6.19 months. This highlights the need to continue measurements at further follow-up periods in the current study to establish the trend in the current study’s results compared to other studies with longer follow-up periods. When comparing the titanium to PEEK, Chen et al. concluded that there were significant differences in overall Cobb angle at follow-up, with PEEK cages maintaining 13.65 ± 8.92° and declining by only 4.84 ± 2.39° from the post-operative imaging [11].

There is a lack of literature looking at the C2-C7 Cobb angle in titanium implants, especially long-term outcomes. A meta-analysis by Zhai et al. compared the differences between 3D‐printed porous titanium and PEEK cages for ACDF [29]. Only six papers were found that reported on the C2-C7 Cobb angle, and Zhai et al. [29] concluded that the post-operative Cobb angle was greater in the titanium group than that of the PEEK group. Furthermore, titanium cages had less loss in C2-C7 Cobb angle, showing more maintenance of cervical lordosis. There is a lack of literature looking at the C2-C7 Cobb angle in titanium implants, especially long-term outcomes. The current study’s hypothesis behind the slight decline in overall C2-C7 Cobb angle over the follow-up period can be explained by osteointegration and settling of the cage into the vertebral bodies. 

There was a statistically significant positive correlation between the change in IDH and the change in overall C2-C7 Cobb angle (p=0.002) in the current study. The relationship between change in IDH and change in segmental Cobb angle was also positively related (p=0.162), although this was not statistically significant. Similarly, Choi et al. [30] also identified a positive correlation between the change in overall IDH and segmental Cobb angle, with a statistical significance of p=0.038. Choi et al. studied 68 patients with degenerative disease who underwent single-level ACDF [30]. Notably, all patients in their study received anterior plating, which is a slight difference in surgical technique compared to the current study. Another methodological difference is that Choi et al. conducted follow-up X-rays only six weeks post-operatively, compared to six months in the current study [30]. In support of the similarity between the methodologies, both studies used the same technique for measuring IDH and segmental Cobb angle, enhancing the accuracy and comparability of the values. Additionally, the same correlation analyses between radiological outcomes were performed in both studies. Another important consideration is the difference in implant materials used: Choi et al. [30] used intervertebral allografts, while the current study used titanium cages. Despite this, the consistency in outcomes between the two studies supports that titanium cages are not inferior to other implant types regarding radiological outcomes.

Strengths and limitations of this study

This retrospective study, conducted in a regional spinal unit, provides comprehensive data on the initial radiological outcomes of CONDUIT^TM^ EIT Cellular Titanium Cages in ACDF procedures, addressing gaps in the current literature regarding new titanium implants. The main limitation of this study is its retrospective nature. Additionally, clinical outcomes were not assessed, preventing comparison alongside radiological outcomes. Although the cohort is small, this implant has only recently been introduced into the BHSCT, and therefore, this study provides valuable initial findings for outcomes of the EIT Cellular Titanium Cage in ACDF surgery. 

## Conclusions

The CONDUIT^TM^ EIT Cellular Titanium Cage demonstrates promising initial radiological outcomes in ACDF procedures. The implant is successful in increasing intervertebral disc height and restoring segmental and overall lordosis to the cervical spine. Future research should incorporate a longer follow-up period and clinical outcomes to provide comprehensive data that can better inform clinical management. Repeating this study in the same trust with PEEK cages would allow for direct comparison between the implant types. Furthermore, performing the study in other regional spinal units would provide additional data to validate results. The initial results from this study suggest that titanium cages show successful initial radiological outcomes and efficacy in ACDF procedures.
